# Hepatitis B Virus-Encoded HBsAg Contributes to Hepatocarcinogenesis by Inducing the Oncogenic Long Noncoding RNA LINC00665 through the NF-κB Pathway

**DOI:** 10.1128/spectrum.02731-21

**Published:** 2022-08-22

**Authors:** Shivaksh Ahluwalia, Belal Ahmad, Uzma Salim, Dipannita Ghosh, Vinay Kamuju, Arpita Ghosh, Khadija Dabeer, Manoj B. Menon, Perumal Vivekanandan

**Affiliations:** a Kusuma School of Biological Sciences, Indian Institute of Technology Delhi, New Delhi, India; b Department of Biosystems Science and Engineering, ETH Zurich, Basel, Switzerland; c CSIR, Institute of Genomics & Integrative Biology, New Delhi, India; d Academy of Scientific & Innovative Research, CSIR Human Resource Development Centre, Ghaziabad, Uttar Pradesh, India; University of Manitoba

**Keywords:** HBsAg, LINC00665, NF-κB, hepatitis B virus, hepatocellular carcinoma, long noncoding RNA, oncogenesis, oncogenic virus, virus-host interactions

## Abstract

Clinical and *in vivo* studies have demonstrated a role for hepatitis B virus (HBV)-encoded HBsAg (hepatitis B surface antigen) in HBV-related hepatocellular carcinoma (HCC); however, the underlying mechanisms remain largely unknown. Here, we investigated the role of HBsAg in regulating long noncoding RNAs (lncRNAs) involved in HCC progression. Our analysis of microarray data sets identified LINC00665 as an HBsAg-regulated lncRNA. Furthermore, LINC00665 is upregulated in liver samples from HBV-infected patients as well as in HCC, specifically in HBV-related HCC liver samples. These findings were supported by our *in vitro* data demonstrating that HBsAg, as well as HBV, positively regulates LINC00665 in multiple HBV cell culture models. Next, we evaluated the oncogenic potential of LINC00665 by its overexpression and CRISPR interference (CRISPRi)-based knockdown in various cell-based assays. LINC00665 promoted cell proliferation, migration, and colony formation but inhibited cell apoptosis *in vitro*. We then identified the underlying mechanism of HBsAg-mediated regulation of LINC00665. We used immunofluorescence assays to show that HBsAg enhanced the nuclear translocation of NF-κB factors in HepG2 cells, confirming that HBsAg activates NF-κB. Inhibition of NF-κB signaling nullified HBsAg-mediated LINC00665 upregulation, suggesting that HBsAg acts through NF-κB to regulate LINC00665. Furthermore, the LINC00665 promoter contains NF-κB binding sites, and their disruption abrogated HBsAg-induced LINC00665 upregulation. Finally, HBsAg facilitated the enrichment of the NF-κB factors NF-κB1, RelA, and c-Rel in the LINC00665 promoter. Taken together, this work shows that HBsAg can drive hepatocarcinogenesis by upregulating oncogenic LINC000665 through the NF-κB pathway, thereby identifying a novel mechanism in HBV-related HCC.

**IMPORTANCE** Hepatitis B virus (HBV) is a major risk factor for hepatocellular carcinoma (HCC). Numerous reports indicate an oncogenic role for HBV-encoded HBsAg; however, the underlying mechanisms are not well understood. Here, we studied the role of HBsAg in regulating lncRNAs involved in hepatocarcinogenesis. We demonstrate that HBsAg, as well as HBV, positively regulates oncogenic lncRNA LINC00665. The clinical significance of this lncRNA is highlighted by our observation that LINC00665 is upregulated in liver samples during HBV infection and HBV-related HCC. Furthermore, we show LINC00665 can drive hepatocarcinogenesis by promoting cell proliferation, colony formation, and cell migration and inhibiting apoptosis. Taken together, this work identified LINC00665 as a novel gene through which HBsAg can drive hepatocarcinogenesis. Finally, we show that HBsAg enhances LINC00665 levels in hepatocytes by activating the NF-κB pathway, thereby identifying a novel mechanism by which HBV may contribute to HCC.

## INTRODUCTION

Hepatocellular carcinoma (HCC is the most prevalent form of liver cancer and a major challenge to health care worldwide (1). Chronic infection with hepatitis B virus (HBV) (CHB) is the predominant risk factor associated with HCC (2). HBV is a small, enveloped virus belonging to the family *Hepadnaviridae*. Its partially double-stranded 3.2-kb genome encodes 4 overlapping open reading frames (ORFs). (i) The precore/core ORF encodes the core protein (capsid) and the secretory HBeAg. (ii) The P ORF encodes the terminal peptides and the HBV polymerase. (iii) The X ORF encodes the oncogenic transactivator hepatitis B X protein (HBx). (iv) Finally, the pre-S1/S ORF encodes 3 proteins: large (pre-S1 protein), middle (pre-S2 protein), and small (HBsAg) surface proteins, which make up the lipid envelope (3). HBx is the most extensively studied HBV-encoded oncogenic factor. Its ability to transactivate host genes directly has been shown to drive hepatocarcinogenesis by dysregulating vital cellular pathways. In contrast, the role of other HBV proteins in HCC development is not well understood.

The ~24-kDa HBsAg is the most abundantly produced HBV protein and is a major constituent of the HBV proteolipid envelope (4). HBsAg, like other surface proteins, is transported to the endoplasmic reticulum (ER) and secreted as part of the both (i) the infective HBV virion and (ii) the abundantly produced noninfective 22-nm subviral particles (5, 6). The presence of HBsAg in serum is used as a diagnostic marker, and the loss of detectable HBsAg in serum (seroclearance) is an established therapeutic endpoint (7, 8). Clinical studies show that the presence and levels of HBsAg in circulation are linked to cirrhosis, progression to HCC, and poor clinical outcome in HBV infection (7, 9–14). Furthermore, HBsAg seroclearance significantly reduces the risk of HCC development (15–17). *In vivo* studies demonstrated that HBsAg transgenic mice spontaneously developed HCC (18). Small-interfering-RNA (siRNA)-mediated knockdown of HBsAg inhibited viral replication and progression to HCC in human HCC mouse models, further highlighting the role of HBsAg in tumorigenesis (19). HBsAg has been reported to alter the functioning of vital cellular pathways – including the unfolded protein response (UPR) pathway, ER-β signaling pathway, and nuclear factor-κB (NF-κB) pathway, among others, thereby contributing to HCC progression (18, 20, 21). These studies support oncogenic functions of HBsAg in HCC progression; however, the underlying mechanisms driving HBsAg-mediated hepatocarcinogenesis remain largely unknown. In this study, we explored the role of HBsAg in regulating long noncoding RNAs (lncRNAs) to better understand its role in HCC development.

Noncoding RNAs which are more than 200 nucleotides in length are classified as lncRNAs. These functional RNA molecules have vital roles in essential cellular processes, including cell cycle regulation, DNA repair, cellular development and differentiation, and host-pathogen interaction, among others (22). A whole-transcriptome study revealed that over 1,000 lncRNAs are differentially expressed in HCC (23). However, only a few of these lncRNAs have been shown to be regulated by HBV. Oncogenic lncRNAs like HULC (24), UCA1 (25), DBH-AS1 (26), ZEB2-AS1 (27), and MALAT1 (28) have been shown to be upregulated by HBV-encoded HBx protein, whereas tumor suppressor lncRNA DREH (29) was observed to be downregulated by the HBx protein. HBV-encoded proteins other than HBx have not been studied for their ability to regulate lncRNAs. Numerous clinicoepidemiological studies support an oncogenic function for HBsAg. This HBV protein has been shown to dysregulate oncogenic and tumor-suppressive genes as well as microRNAs (miRNAs); however, its role in regulating lncRNAs has not been explored (18, 19, 30).

In the current study, we sought to understand the role of HBsAg in regulating lncRNAs involved in hepatocarcinogenesis. We analyzed multiple gene expression data sets deposited in the Gene Expression Omnibus (GEO) and identified LINC00665 as an HBsAg-regulated lncRNA that is upregulated during HBV-related HCC. We confirmed these findings *in vitro* by demonstrating that LINC00665 is positively regulated by HBsAg, as well as by HBV, in various cell culture models. Next, we evaluated the oncogenic functions of LINC00665 using various *in vitro* cell-based assays in gain-of-function and loss-of-function studies. We further wanted to decipher the mechanism by which HBsAg regulates LINC00665. LINC00665 has been previously shown to be upregulated by NF-κB signaling in hepatocytes (31). Furthermore, numerous clinical, *in vivo*, and *in vitro* studies have demonstrated that HBsAg activates NF-κB signaling (21, 32, 33). Based on these findings, we hypothesized that HBsAg regulates LINC00665 through the NF-κB pathway. We tested this hypothesis by evaluating the effect of an NF-κB inhibitor on the HBsAg-mediated regulation of LINC00665 transcription and LINC00665 promoter activity. We further tested the role of NF-κB binding sites in the HBsAg-mediated regulation of LINC00665 promoter activity. Finally, we used binding assays to assess whether HBsAg promotes the binding of NF-κB factors at the LINC00665 promoter.

## RESULTS

### LINC00665 is positively regulated by HBsAg and is upregulated in HBV-related HCC.

**FIG 1:**
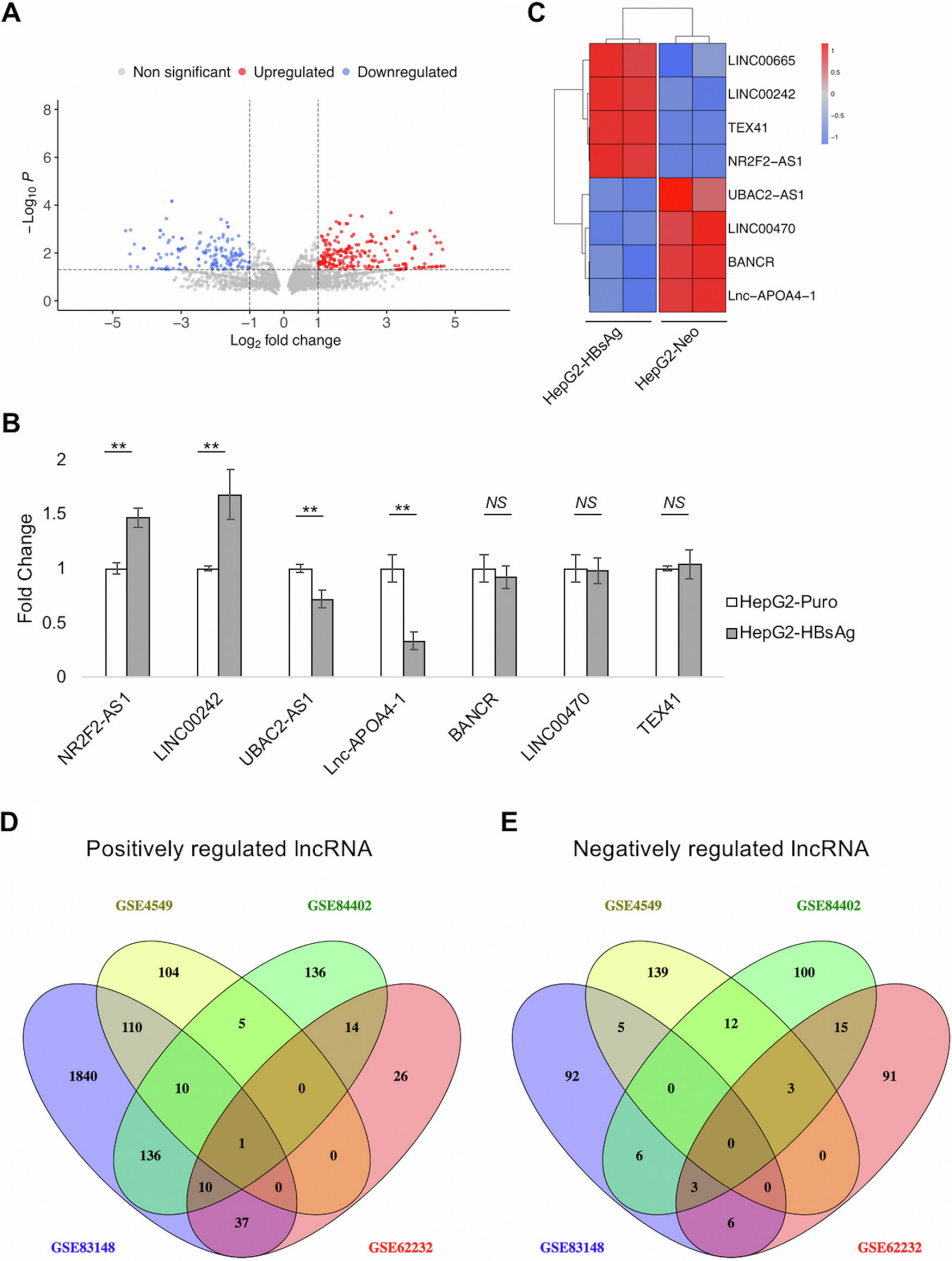
LINC00665 is an HBsAg-regulated lncRNA which is upregulated during HBV infection and HCC, specifically HBV-related HCC. Four microarray data sets were extracted from GEO and analyzed to identify HBsAg-regulated lncRNAs which are involved in HBV-related HCC. (A) Upregulated and downregulated lncRNAs (*P* < 0.05 and |log_2_ FC|> 1) are graphically represented in a volcano plot for data set GSE4549 (HepG2-HBsAg versus control HepG2-Neo cells). (B) Seven of these lncRNAs were randomly chosen, and qPCR was performed in HepG2-HBsAg and control HepG2-Puro cells to validate microarray analysis for GSE4549. **, *P* < 0.01; NS, not significant (paired Student’s *t* test). (C) Comparison of these qPCR data with the cDNA microarray heat map (GSE4549) for these 7 randomly selected lncRNAs (and LINC00665) demonstrated that 4 of 7 lncRNAs showed an expression profile similar to that in GSE4549. Furthermore, DElncRNAs (*P* < 0.05) were identified in HBV and HCC-related clinical studies (GSE84402, GSE83148, and GSE62232; see the text for details). (D and E) Venn diagrams representing the intersection of (D) positively regulated and (E) negatively regulated lncRNAs in these clinical data sets, along with GSE4549. LINC00665 was the only common DElncRNA among the four data sets. All data are means and standard deviations (SD) from three independent experiments (*n* = 3).

We extracted and analyzed microarray data sets deposited on GEO (https://www.ncbi.nlm.nih.gov/geo/) to identify HBsAg-regulated, clinically relevant lncRNAs. All microarrays analyzed in this study were performed on the GPL570 platform (Affymetrix human genome U133 Plus 2.0 array), which contains 6,189 probes orresponding to 5,149 unique lncRNAs (listed in Data Set S1 in the supplemental material). We first analyzed the microarray data set GSE4549 (34), which comprises the expression data for HepG2 cells stably expressing HBsAg compared to control HepG2-Neo cells (*n* = 2). Our analysis using the R package limma (Bioconductor) revealed a total of 329 differentially expressed lncRNAs (DElncRNAs) (*P* < 0.05 and |log_2_ FC|> 1; FC is fold change), of which 159 were downregulated and 233 were upregulated by HBsAg (Fig. 1A; list in Data Set S2).

To experimentally validate the results of the microarray, we developed HepG2 cells stably expressing HBsAg (HepG2-HBsAg cells) using retroviral transductions as detailed in Materials and Methods. Stable expression of HBsAg in HepG2-HBsAg cells was confirmed by enzyme-linked immunosorbent assay (ELISA) (Fig. S1). Seven DElncRNAs were randomly selected, and quantitative PCR (qPCR) was performed in HepG2-HBsAg cells relative to control HepG2-Puro cells. Four of the seven lncRNAs showed an expression profile similar to that observed in the microarray (Fig. 1B). A heat map depicting the expression of these lncRNA (along with LINC00665) in the data set GSE4549 is provided (Fig. 1C).

Next, we analyzed gene expression in liver samples from HCC (including HBV-related HCC) or HBV-infected patients in the GEO data sets GSE84402 (35), GSE83148 (36), and GSE62232 (37), with the objective of discovering HBsAg-regulated lncRNAs that are dysregulated during HCC (*P* < 0.05). GSE84402 contains the expression profile of HCC tissues and corresponding noncancerous tissues (*n* = 14), in which we identified 500 DElncRNAs (343 upregulated and 157 downregulated; listed in Data Set S3). GSE83148 provides information on the expression profile of HBV-infected livers (*n* = 122) with respect to healthy uninfected livers (*n* = 6), in which we identified 2,558 DElncRNAs (2,463 upregulated and 125 downregulated; listed in Data Set S4) which may be altered by HBV infection. The results were also corroborated with data set GSE62232, containing the expression profile of HCC tissues along with the information on their etiologies. Here, we compared the gene expression profile of HBV-related HCC (*n* = 10) to that of alcohol-induced or primary HCC (*n* = 37) (38) and identified 258 DElncRNAs (122 upregulated and 136 downregulated; listed in Data Set S5) that are specifically dysregulated in HBV-related HCC and not in other forms of HCC. DElncRNAs common to all four data sets are likely to be dysregulated in the presence of HBsAg (from GSE4549) during HBV infection (from GSE83148) and during HCC (GSE84402), more specifically in HBV-related HCC (GSE62232). We observed only one DElncRNA, LINC00665, that was upregulated in all 4 GEO data sets (Fig. 1D), whereas no negatively regulated lncRNA was common to all four studies (Fig. 1E). Box plots for LINC00665 expression in the liver samples from HCC or HBV-related data sets are presented in Fig. S2. Taken together, this analysis indicated that LINC00665 is upregulated by HBsAg, during HBV infection and in HBV-related HCC.

### HBsAg and HBV positively regulate LINC00665 in cell culture.

**FIG 2:**
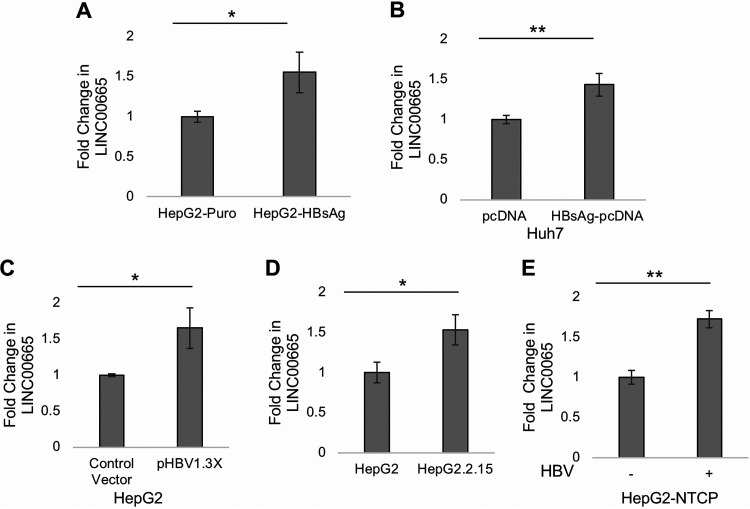
HBsAg and HBV positively regulate LINC00665 in different cell culture models. Real-time PCR analysis showed that LINC00665 expression was elevated in the presence of HBsAg in (A) stable HepG2-HBsAg cells relative to control HepG2-Puro cells and (B) Huh7 cells transiently transfected with HBsAg-pcDNA relative to control vector (pcDNA) for 48 h. Additionally, LINC00665 was upregulated in three different HBV cell culture models. (C) HepG2 cells were transiently transfected with the 1.3× HBV genome construct or control vector, and qPCR was performed 48 h after transfection in the transfection model. (D) In the integrated HBV model, LINC00665 levels were measured in HepG2.215 cells relative to HepG2 control cells. (E) Finally, HBV derived from HepG2.2.15 cells were used to infect HepG2-NTCP cells in the infection model as described in Materials and Methods. LINC00665 levels were determined in the presence or absence of infective HBV. All data are means and SD from three independent experiments (*n* = 3). *, *P* < 0.05; **, *P* < 0.01 (paired Student’s *t* test).

To test our *in silico* findings, we evaluated whether HBsAg regulates LINC00665 in cell culture. Quantitative PCR showed an ~1.5-fold increase in LINC00665 expression in HepG2 cells stably expressing HBsAg (HepG2-HBsAg) relative to control HepG2-Puro cells (Fig. 2A). Furthermore, we also observed that LINC00665 expression was significantly increased in Huh7 cells transiently transfected with HBsAg-pcDNA relative to pcDNA (Fig. 2B).

In addition to HBsAg, we also assessed whether the LINC00665 expression was altered in the presence of the HBV whole genome. To that end, qPCR was performed to evaluate LINC00665 expression in the presence of HBV in 3 different cell culture models: (i) an HBV transfection model in which HepG2 cells were transfected with the 1.3× pHBV construct (Fig. 2C), (ii) an HBV genome integration model in which HepG2.2.15 cells carry integrated copies of the HBV genome (Fig. 2D), and (iii) an HBV infection model in which HepG2-NTCP cells were infected with HBV purified from the supernatant of HepG2.2.15 cells (Fig. 2E). We observed that HBV significantly enhances LINC00665 expression in each of the three HBV cell culture models, similar to the levels observed in the presence of HBsAg.

### LINC00665 overexpression increases cell proliferation, colony formation, and migration and inhibits apoptosis *in vitro*.

**FIG 3:**
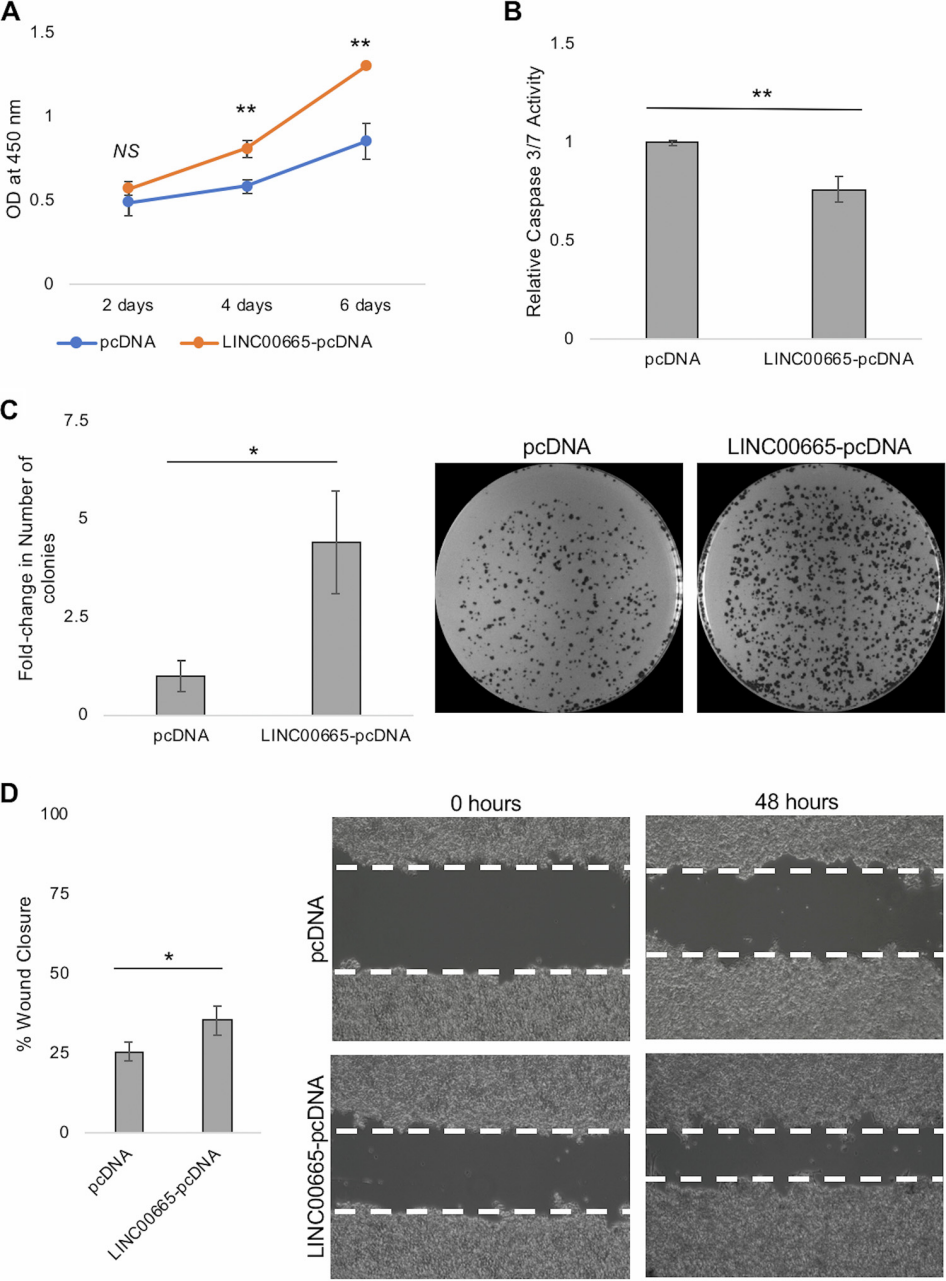
LINC00665 overexpression increases cell proliferation, colony formation and cell migration, but inhibits apoptosis. Cell-behavior assays were performed in HepG2 cells transfected with LINC00665-pcDNA or pcDNA (control) as described in the methods section. (A) Cell proliferation was detected using CCK-8 solution, 2, 4 and 6 days after transfection. (B) Apoptosis was determined by measuring caspase 3/7 activity 48 h after transfection. Caspase 3/7 activity was normalized to that of pcDNA (control) transfected cells. (C) A clonogenic assay was performed by growing transfected HepG2 cells for 2 weeks, after which the colonies were stained and counted. The number of colonies were normalized to those in pcDNA control and is represented graphically as the fold change in the number of colonies. (D) Cell migration was detected by scratch assay. The percent wound closure is represented graphically and expresses the percentage of the area of the scratch that was covered in 48 h. All data are means and SD from three independent experiments (*n* = 3). *, *P* < 0.05; **, *P* < 0.01; NS, not significant (paired Student’s *t* test).

We demonstrated that LINC00665 is positively regulated in the presence of HBsAg, as well as HBV *in vitro*. Furthermore, our *in silico* analysis suggests that LINC00665 expression is elevated in HBV-related HCC. To better understand the role of LINC00665 in HCC development, we (i) inhibited LINC00665 using CRISPR interference (CRISPRi) and (ii) overexpressed LINC00665 by transient transfection. For gain-of-function studies, the predominant LINC00665 transcript was cloned into a mammalian expression vector (LINC00665-pcDNA) (31), and cell behavior was assessed relative to empty vector (pcDNA) in HepG2 cells. We observed that overexpression of LINC00665 significantly increased proliferation of HepG2 cells (Fig. 3A). Furthermore, LINC00665 overexpression significantly inhibited apoptosis as observed by decreased caspase 3/7 activity in HepG2 cells (Fig. 3B). Finally, LINC00665 overexpression increased colony formation in the clonogenic assay (Fig. 3C), as well as cell migration as observed by increased wound closure in the scratch assay (Fig. 3D).

### LINC00665 knockdown inhibits cell proliferation, colony formation, and migration and induces apoptosis *in vitro*.

**FIG 4:**
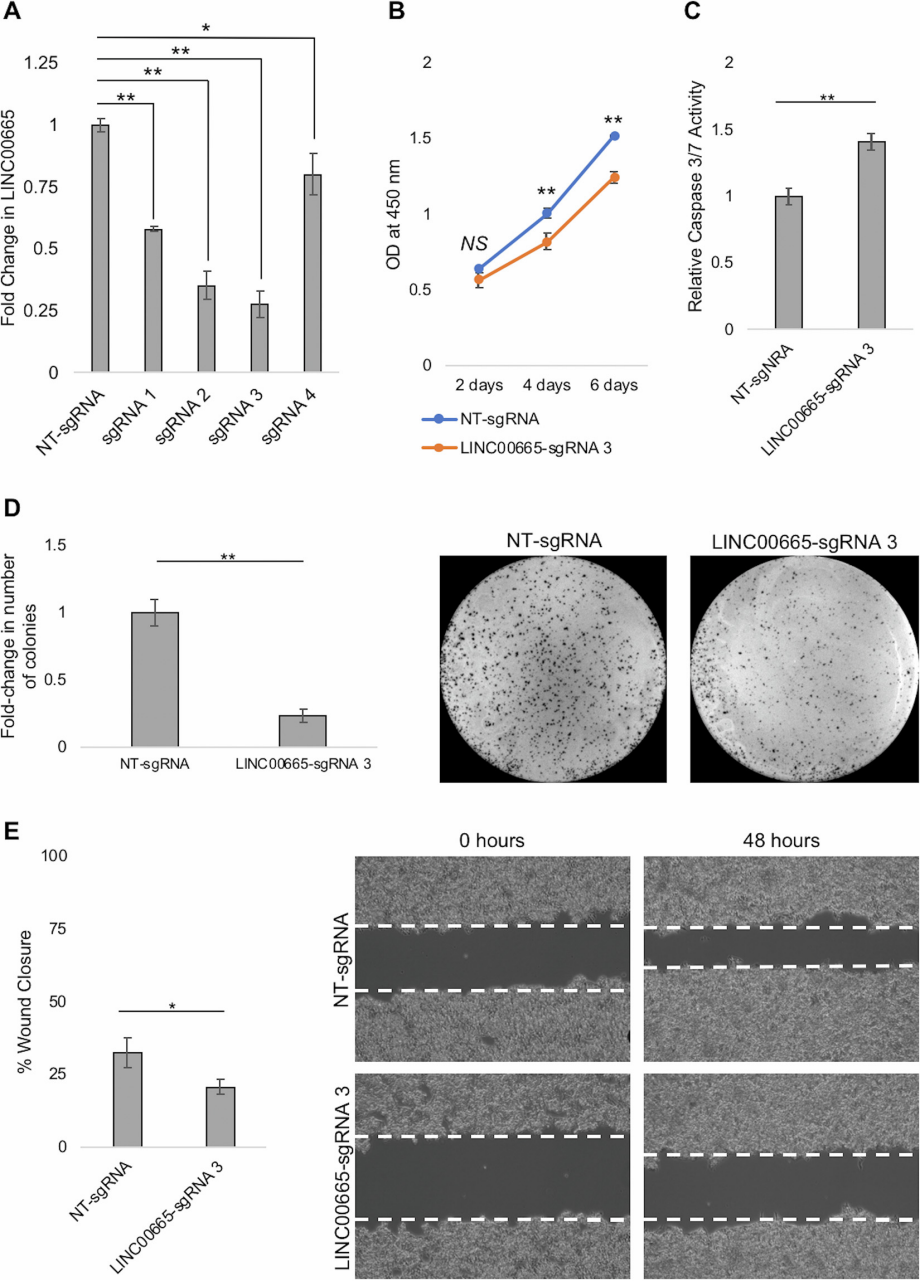
LINC00665 knockdown inhibits cell proliferation, colony formation, and cell migration, but induces apoptosis. (A) Four sgRNAs targeting the LINC00665 promoter were screened for their ability to knockdown LINC00665 by CRISPRi. The sgRNAs were cloned separately into the MLM3636 expression vector and transfected into HepG2-dCas9-KRAB cells. qPCR analysis showed that LINC00665-sgRNA 3 knocked down the lncRNA by ~75%. Cell behavior assays were performed in HepG2-dCas9-KRAB cells transfected with LINC00665-sgRNA 3 or NT-sgRNA (control). (B) Cell proliferation was assayed using CCK-8 solution, 2, 4, and 6 days after transfection. (C) Apoptosis was determined by measuring caspase 3/7 activity 48 h after transfection. Caspase 3/7 activity was normalized to that of control NT-sgRNA-transfected cells. (D) A clonogenic assay was performed by growing sgRNA-transfected cells for 2 weeks, after which the colonies were stained and counted. The number of colonies were normalized to those in the NT-sgRNA control and are graphically represented. (E) Cell migration was determined by measuring percentage of the wound area covered in 48 h in a scratch test. All data are means and SD from three independent experiments (*n* = 3). *, *P* < 0.05; **, *P* < 0.01; NS, not significant (paired Student’s *t* test).

For CRISPRi-based knockdown of LINC00665, we developed HepG2 cells stably expressing the KRAB (Krüppel-associated box) repressor fused to dCas9 (see Materials and Methods for details) (Fig. S3). Four spacer sequences targeting regions proximal to the LINC00665 transcription start site (TSS) were cloned into a single guide RNA (sgRNA) expression vector (MLM3636). These four clones were transfected individually into HepG2-dCas9-KRAB cells to assess the ability of each sgRNA to knockdown LINC00665. Quantitative PCR analysis showed that all 4 sgRNAs significantly knocked down LINC00665; however, maximum inhibition (~75%) was achieved by LINC00665 sgRNA 3 (Fig. 4A). Hence, further loss-of-function studies were performed using LINC00665 sgRNA 3, while a nontargeting scrambled sgRNA (NT-sgRNA) was used as control. We observed that LINC00665 knockdown significantly inhibited cell proliferation 4 and 6 days after transfection (Fig. 4B), colony formation (Fig. 4D), and cell migration (Fig. 4E) in HepG2 cells. Furthermore, inhibition of LINC00665 significantly enhanced apoptosis *in vitro* (Fig. 4C). Taken together, these results (Fig. 3 and 4) demonstrate that LINC00665 promotes cell proliferation, colony formation, and cell migration while inhibiting apoptosis. Furthermore, these results indicate that HBsAg (and HBV)-induced expression of the oncogenic lncRNA LINC00665 can contribute to HCC development.

### HBsAg acts through the NF-κB pathway to regulate LINC00665 expression.

**FIG 5:**
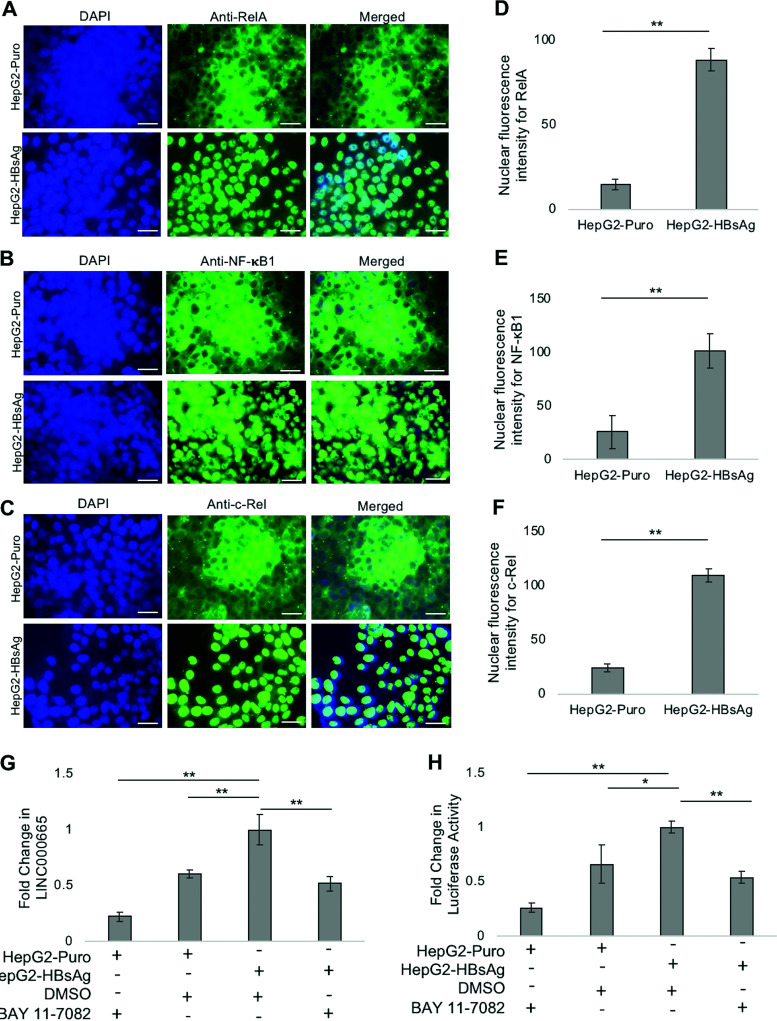
HBsAg regulates LINC00665 through the NF-κB pathway. An immunofluorescence assay was performed to visualize (A) RelA, (B) NF-κB1, and (C) c-Rel in stable HepG2-HBsAg cells and control HepG2-Puro cells as described in Materials and Methods. Nuclear fluorescence intensity of (D) RelA, (E) NF-κB1, and (F) c-Rel was significantly greater in HepG2-HBsAg than in control HepG2-Puro cells. Bar, 10 μm. (G) Real-time PCR was performed to measure change in LINC00665 expression in HepG2-HBsAg and HepG2-Puro cells in the presence of BAY 11-7082 (2 μM) or vehicle control (DMSO). Fold change in LINC00665 expression was measured relative to HepG2-HBsAg cells treated with DMSO. (H) The LINC00665 minimal promoter was cloned upstream of the luciferase reporter gene in the PGL3-basic vector. This construct was used to transfect HepG2-HBsAg and HepG2-Puro cells treated with BAY 11-7082 (2 μM) and vehicle control (DMSO). A luciferase assay was performed to measure the change in LINC00665 promoter activity 48 h after transfection. Fold change in LINC00665 promoter activity was normalized to that of HepG2-HBsAg cells treated with DMSO. Addition of the NF-κB inhibitor BAY 11-7082 nullified the HBsAg-mediated upregulation of LINC00665. All data are means and SD from three independent experiments (*n* = 3). *, *P* < 0.05; **, *P* < 0.01; NS, not significant (paired Student’s *t* test).

Next, we wanted to elucidate the mechanism by which HBsAg positively regulates LINC00665 expression. It has been shown that LINC00665 is an NF-κB-responsive lncRNA (31). Additionally, studies have shown HBsAg induces NF-κB signaling in clinical samples, mouse models, and cell culture (21, 32, 33). Based on these findings, we investigated whether HBsAg-induced NF-κB signaling could regulate LINC00665 expression. To test our hypothesis, we first wanted to evaluate whether HBsAg activates NF-κB signaling in HepG2-HBsAg cells used in this study. The NF-κB transcription factors, namely, NF-κB1 (p105 and p50), NF-κB2 (p100 and p52), RelA (p65), c-Rel, and RelB, are sequestered within the cytoplasm in the absence of NF-κB activators. Activating stimuli induce a signaling cascade leading to the translocation of NF-κB factors into the nucleus (39). To test whether HBsAg can activate NF-κB signaling, leading to enhanced nuclear localization of NF-κB factors, we performed a direct immunofluorescence assay. This was done using DAPI (4′,6-diamidino-2-phenylindole) for nuclear staining and antibodies targeting the NF-κB factors NF-κB1, RelA, and c-Rel in HepG2-HBsAg cells and control HepG2-Puro cells. We observed that nuclear localization of RelA (Fig. 5A), NF-κB1 (Fig. 5B), and c-Rel (Fig. 5C) was greater in HepG2-HBsAg than in control HepG2-Puro cells, as detected by fluorescence microscopy. The nuclear fluorescence intensity for these NF-κB factors was significantly greater in HepG2-HBsAg than in control HepG2-Puro cells (Fig. 5D to F). In addition, HepG2-HBsAg cells showed higher levels of NF-κB target genes IL1A and GPC6 than HepG2-Puro cells (Fig. S4). These findings are in accordance with previous reports that linked HBsAg to NF-κB signaling (21, 32, 33).

Next, we used BAY 11-7082, an NF-κB inhibitor, to evaluate the role of the NF-κB pathway in HBsAg-mediated regulation of LINC00665. HepG2-HBsAg and HepG2-Puro (control) cells were treated with BAY 11-7082 or vehicle control (dimethyl sulfoxide [DMSO]), as indicated (Fig. 5G), and its effect on LINC00665 expression was measured by qPCR. We observed that the addition of BAY 11-7082 significantly reduced LINC00665 levels in HepG2-Puro cells, substantiating previous reports showing that LINC00665 is regulated by the NF-κB pathway (31). As shown in Fig. 2, the presence of HBsAg increased LINC00665 expression. However, the addition of BAY 11-7082 to HepG2-HBsAg nullified this HBsAg-mediated upregulation of LINC00665, indicating a role for NF-κB signaling in the HBsAg-mediated activation of LINC00665 expression. To corroborate these findings, we evaluated the effect of BAY 11-7082 on the LINC00665 promoter in the presence of HBsAg. We cloned the LINC00665 minimal promoter upstream of the luciferase gene in the PGL3-basic vector as described in Materials and Methods. This reporter construct was transfected into HepG2-HBsAg and HepG2-Puro cells in the presence of BAY 11-7082 or vehicle control (DMSO). Luciferase assay demonstrated that the presence of HBsAg significantly increased the LINC00665 promoter activity, suggesting that HBsAg acts at the transcriptional level to enhance LINC00665 expression (Fig. 5H). Finally, the NF-κB inhibitor (BAY 11-7082) suppressed the HBsAg-induced activation of the LINC00665 promoter. Taken together, these findings demonstrate that HBsAg facilitates LINC00665 transcription via the NF-κB pathway.

### HBsAg acts through κB sites and enriches NF-κB factors at the LINC00665 promoter.

**FIG 6:**
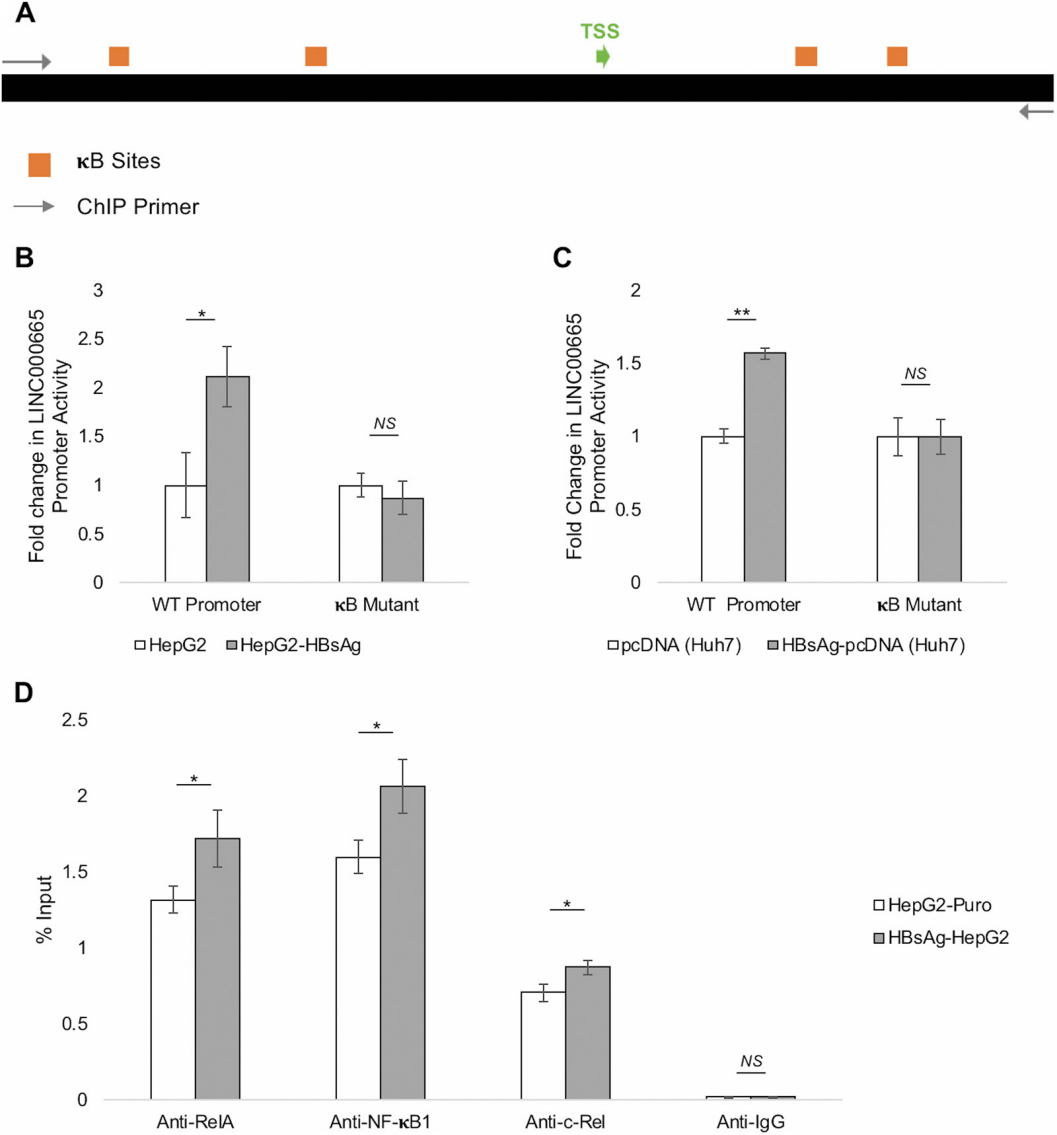
HBsAg induced NF-κB transcription factors bind and activate the LINC00665 promoter through κB sites. (A) Illustration depicting the position of κB site clusters (see Table S2 for details) with respect to the TSS in the LINC00665 promoter and the position of the primer pair used for ChIP. Mutations were introduced to disrupt these κB sites. (B and C) A luciferase assay was performed to measure the activity of the wild-type LINC00665 minimal promoter (WT promoter) and LINC00665 promoter with disrupted κB sites (κB mutant) in the presence of HBsAg in (B) stable HepG2-HBsAg relative to control HepG2-Puro cells and in (C) Huh7 cells transiently transfected with HBsAg-pcDNA or pcDNA control. Disrupting NF-κB sites abrogated HBsAg-induced activation of the LINC00665 promoter. (D) ChIP-qPCR was performed after extracting chromatin from HepG2-HBsAg and HepG2-Puro cells, using anti-NF-κB1, anti-c-Rel, and anti-RelA antibodies. HBsAg promoted the binding each of the NF-κB factors at the LINC00665 promoter in HepG2-HBsAg cells relative to control HepG2-Puro cells. Rabbit IgG was used as a negative control. All data are means and SD from three independent experiments (*n* = 3). *, *P* < 0.05; **, *P* < 0.01; NS, not significant (paired Student’s *t* test).

NF-κB factors coordinate into homo- or heterodimers at DNA motifs known as κB sites within promoters/regulatory elements of target genes to modulate their activity (39). To further gain insights into the mechanism by which HBsAg-activated NF-κB pathway regulates LINC00665, we investigated the role of κB sites in the LINC00665 promoter and their interaction with NF-κB transcription factors in the presence of HBsAg. NF-κB transcription factor binding sites in the LINC00665 promoter were identified using methods described previously (31, 40–42) (Fig. 6A) (see Table S2 for details on κB sites). Mutations were designed to disrupt these κB sites to study their role in HBsAg-mediated regulation of LINC00665 via NF-κB. The wild-type (WT) LINC00665 minimal promoter and the LINC00665 promoter with disrupted κB sites (κB-mutant LINC00665 promoter) were cloned separately upstream of the luciferase reporter in the PGL3-basic vector (see Materials and Methods for details). A luciferase assay was performed to compare the responses of these promoters in HepG2-HBsAg cells stably expressing HBsAg (Fig. 6B) and Huh7 cells transiently transfected with HBsAg-pcDNA (Fig. 6C). As observed in this study, HBsAg significantly enhanced the activity of the wild-type LINC00665 promoter in cells stably or transiently expressing HBsAg. However, there was no significant change in the activity of the mutant LINC00665 promoter (with disrupted κB sites) in either of the cell lines expressing HBsAg. These results show that disrupting κB sites in the LINC00665 promoters abrogates HBsAg-mediated activation of LINC00665 expression.

Next, we assessed the enrichment of NF-κB factors at the LINC00665 promoter in the presence of HBsAg by chromatin immunoprecipitation (ChIP). Chromatin from HepG2-HBsAg cells and HepG2-NTCP cells was extracted, and DNA bound to NF-κB1, RelA, and c-Rel was immunoprecipitated using their targeting antibodies. Rabbit IgG antibody was used as a negative control. Quantitative PCR was performed as described in Materials and Methods using primer pairs flanking κB sites in the LINC00665 promoter (Fig. 6A). We observed a significant increase in binding of NF-κB1, cRel, and Rel-A at the LINC00665 promoter in the presence of HBsAg (Fig. 6D). Collectively, these results demonstrate that HBsAg acts through κB sites in the LINC00665 promoter and HBsAg-activated NF-κB signaling leads to enrichment of NF-κB factors at the LINC00665 promoter.

### HBsAg does not affect the stability of LINC00665.

We then asked whether HBsAg can directly or indirectly affect the stability of LINC00665. The HBx protein, for example, can stabilize host mRNA by upregulating RNA-binding proteins (43). HBsAg expression did not affect the stability of LINC00665 following transcriptional inhibition with actinomycin D in HepG2 cells (Fig. S5). Taken together, our results indicate that HBsAg-mediated increase in LINC00665 is primarily associated with the activation of NF-κB signaling, resulting in the enrichment of NF-κB factors at the LINC00665 promoter.

## DISCUSSION

**FIG 7:**
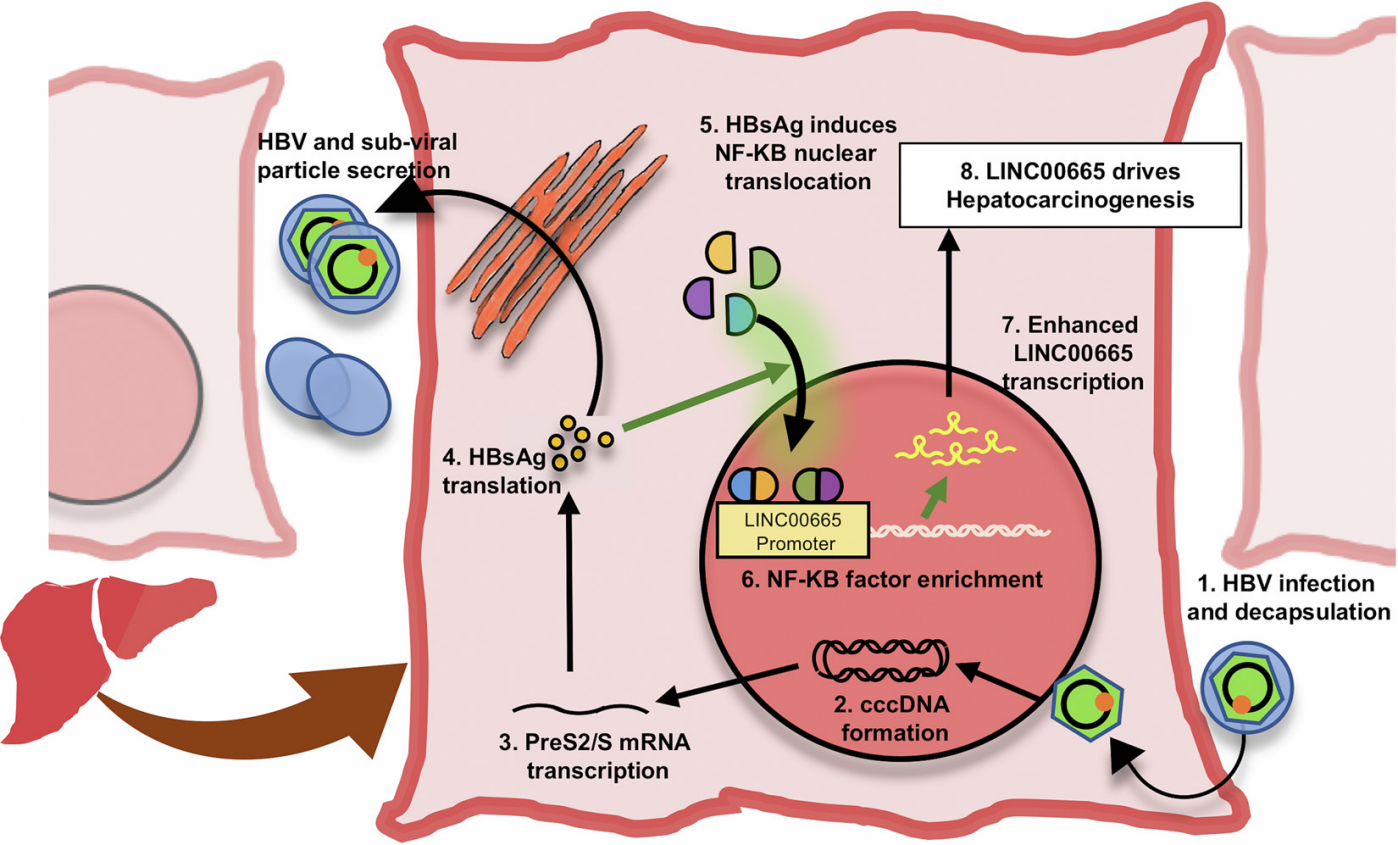
HBsAg-induced NF-κB factors bind and upregulate LINC00665 expression. In the course of HBV infection, the virus enters hepatocytes and undergoes nucleocapsid disassembly to release the HBV genome into the nucleus. Here, the HBV genome is acted upon by host factors to form the transcriptionally active covalently closed circular DNA (cccDNA). HBV cccDNA transcribes the HBV mRNA, including the pre-S2/S 2.1-kb mRNA, which in turn is translated to HBsAg and pre-S2 protein. HBsAg is the major component of the HBV envelope and is secreted as both infective HBV particles and noninfective subviral particles. In the current study, we show that HBsAg induces nuclear translocation of the NF-κB factors NF-κB1, RelA, and c-Rel. These factors bind and activate the LICN00665 promoter, leading to increased transcription of the lncRNA. Using various cell-based assays, we show LINC00665 drives hepatocarcinogenesis by promoting cell proliferation, colony formation, and cell migration but inhibiting apoptosis.

Clinical and *in vivo* studies indicate that HBsAg promotes HCC development; however, the underlying mechanisms remain poorly understood (7, 9–14, 18). Here, we investigated the role of HBsAg in regulating lncRNAs and tested whether HBsAg-regulated lncRNAs can drive hepatocarcinogenesis. Our analysis of publicly available microarray data from hepatic cells overexpressing HBsAg, as well as gene expression data from liver samples, identified LINC00665 as an HBsAg-regulated lncRNA which is upregulated during HBV infection as well as HBV-related HCC (Fig. 1). The ability of HBsAg to regulate lncRNAs has not been previously reported. This finding was supported by our *in vitro* data which demonstrated that LINC00665 expression was upregulated in the presence of HBsAg, as well as during HBV replication in 3 different HBV cell culture models (Fig. 2). Next, we performed multiple cell behavior assays and showed that LINC00665 promotes cell proliferation, colony formation, and cell migration but inhibits apoptosis *in vitro* (Fig. 3 and 4). These findings corroborate previous reports which demonstrate the hepatocarcinogenic function of LINC00665 *in vitro*, as well as *in vivo* (31, 44, 45). Taken together, these results identify a novel lncRNA through which HBV, specifically HBsAg, can drive hepatocarcinogenesis in HBV-related HCC. Furthermore, we elucidated the mechanism by which HBsAg regulates LINC00665. We showed that HBsAg acts through κB sites in the LINC00665 promoter to regulate LINC00665 (Fig. 5B and C). We further show that (i) HBsAg promotes nuclear translocation of RelA, NF-κB1, and c-Rel (Fig. 5) and (ii) HBsAg facilitates an enrichment of these NF-κB factors at the LINC00665 promoter (Fig. 6D). Taken together, these data suggest that HBsAg positively regulates oncogenic LINC00665 through the NF-κB pathway, thereby identifying a novel mechanism by which HBsAg drives hepatocarcinogenesis (Fig. 7).

Chronic liver injury induces uncontrolled inflammation, which is central to liver cirrhosis and HCC development (46, 47). NF-κB is the master regulator of inflammation; hence, aberrant NF-κB activation is frequently observed during chronic inflammation and is closely linked to HCC progression (46, 48, 49). Numerous reports suggest that in addition to canonical NF-κB-activating stimuli, NF-κB can be aberrantly activated by HBV-encoded proteins, thereby inducing hepatocarcinogenesis (32, 33, 50–55). Observations in our study support the role of HBsAg in this model of HBV-driven carcinogenesis through the NF-κB pathway. We demonstrate that HBsAg activates NF-κB signaling and upregulates oncogenic LINC00665 transcription, which can contribute to HCC development.

Clinical data suggest that increased LINC00665 expression is linked to poor disease prognosis and low survival rate in HCC patients (31, 44, 45). LINC00665 is positively regulated by classical and nonclassical NF-κB activators (31), as well as by HBV (HBsAg)-mediated activation of NF-κB signaling (Fig. 1 and 2). In addition to LINC00665, numerous other oncogenes and tumor-suppressive genes have been shown to been dysregulated by aberrant NF-κB signaling (56). Collectively, these findings advocate further studies on the use of NF-κB inhibitors to improve clinical outcomes during HCC. Various NF-κB inhibitors have been explored for their use in HCC therapy; however, these inhibitors have shown limited success in clinical trials. This can be explained by reports suggesting that the role of NF-κB in hepatocarcogenesis is dependent on the cell type (parenchymal or nonparenchymal) and degree of NF-κB inhibition or activation (47, 57). Considering these challenges, it may relevant to develop therapies targeting the downstream genes and pathways dysregulated by aberrant NF-κB signaling, such as LINC00665 and S100A9, among others, rather than employing pan-inhibitory NF-κB strategies in HBV-related HCC (54).

In the natural course of HBV infection, the majority of the ~250 million people chronically infected with HBV enter the inactive carrier state (58). This is characterized by HBsAg seropositivity, suppressed HBV replication, and minimal necroinflammation. The data on liver disease state in these inactive CHB carriers are limited, since liver biopsies are not recommended for these patients; however, cirrhosis and HCC development have been observed in these cases (58). It is important to understand the unique molecular mechanisms governing the liver disease state in inactive carriers, specifically the potential role of HBsAg, which continues to be expressed in these patients while other oncogenic factors are absent. Our findings demonstrating the oncogenic potential of HBsAg through the LINC00665/NF-κB axis may potentially explain the occurrence of HCC in inactive HBV carriers. Hence, our findings also advocate further studies on testing LINC00665 as a biomarker in CHB, specifically in inactive carriers.

In summary, we identify LINC00665 as an HBsAg-regulated clinically relevant lncRNA, dysregulated in HBV-related HCC. We show that LINC00665 is positively regulated by HBsAg and HBV in multiple cell culture models. We demonstrate the oncogenic function of this lncRNA in various cell behavior assays. Finally, our data suggest that HBsAg acts through the NF-κB signaling pathway to enhance LINC00665 transcription. This work (i) identifies a novel mechanism by which HBV-host interactions regulate lncRNAs involved in HCC progression; (ii) demonstrates that HBsAg drives hepatocarcinogenesis through the NF-κB/LINC00665 axis, thereby providing new mechanistic insights into the role of HBsAg in HCC (Fig. 7); (iii) explains, in part, clinical and *in vivo* observations supporting the role of HBsAg in HCC development, specifically in HBV inactivity; and (iv) supports previous studies advocating the use of LINC00665 as a therapeutic and prognostic marker in HCC (31, 44, 45).

## MATERIALS AND METHODS

### Cell culture and transfection.

HepG2, HepG2.2.15, and Huh7 cells were maintained in high-glucose Dulbecco’s modified Eagle medium (Gibco, 11965092) containing 10% fetal bovine serum (FBS; Gibco, 10270106), penicillin-streptomycin solution at 100 U/mL each (Gibco), and 2 mM l-glutamine (Himedia Laboratories Pvt Ltd.). The cells were grown in a humidified incubator at 37°C, in the presence of 5% CO_2_. HepG2-hNTCP-C4 cells were maintained in Dulbecco’s modified Eagle medium/F-12 (Gibco, 10565018) containing 10 mM HEPES (Gibco, 15630080), 200 U/mL penicillin, 200 μg/mL streptomycin, 10% FBS (Gibco, 16000044), 50 μM hydrocortisone (Sigma, H0888), 5 μg/mL insulin (Gibco,12585014), and 400 μg/mL G418 (Himedia Laboratories Pvt Ltd.) (59). Transient transfection of plasmids was performed using Lipofectamine 2000 (Invitrogen) in Opti-MEM (Gibco, 31985070) as per the manufacturer’s protocol. BAY 11-7082 (19542-67-7; Calbiochem, Sigma) used for NF-κB inhibition was kindly provided by Soumen Basak (National Institute of Immunology, New Delhi, India).

### Plasmid constructs and cloning.

The 1.3× HBV genome (HBV genotype D) cloned in pSLIRES-11 (pHBV-1.3×) was kindly provided by Syed N. Kazim (Jamia Millia Islamia, New Delhi, India). The region corresponding to HBsAg (small HBsAg: nucleotides 153 to 833, per the HBV sequence available at GenBank ID V01460.1) was amplified by PCR from the pHBV-1.3× construct. The amplicon was cloned between EcoRI and SalI (New England Biolabs) restriction sites in the pBABE-Puro vector for generation of retroviral particles used for making HepG2-HBsAg cells. Similarly, PCR-amplified HBsAg was cloned between XhoI and KpnI (New England Biolabs) restriction sites in the pcDNA3.1(+) vector (Invitrogen, V790-20) for transient expression of HBsAg.

Double-stranded DNA fragments encoding the major transcript of LINC00665 (31) were synthesized and cloned between KpnI and HindII sites in the pcDNA3.1(+) vector by GeneArt Strings, Thermo Fisher Scientific.

sgRNA target sequences in the LINC00665 minimal promoter were identified using CHOPCHOP (https://chopchop.cbu.uib.no/) (60) (Table S1). For creation of sgRNA expression constructs, complementary single-stranded oligonucleotides containing the sgRNA targeting sequence were annealed and cloned between BsmBI sites in the MLM3636 vector (61, 62). MLM3636 was a gift from Keith Joung (Addgene plasmid number 43860).

Double-stranded DNA fragments corresponding to the LINC00665 minimal promoter (−333 to +250 relative to the TSS) and the LINC00665 minimal promoter with disrupted κB sites was synthesized (GeneArt Strings; Thermo Fisher) and cloned between KpnI and XhoI restriction sites in the PGL3-basic vector. Refer to Table S3 for all oligonucleotides and PCR primers used in creation of plasmid constructs.

### Generation of a dCas9-KRAB-mCherry stable HepG2 cell line.

Lenti-HEK cells (Clontech) were cultured in Dulbecco’s modified Eagle medium (Gibco) supplemented with 10% FBS (Gibco) for the generation of lentivirus particles. The envelope, packaging, and transfer plasmids used for the generation of the virus particles were pMD2.G (Addgene ID 12259), psPAX2 (Addgene ID 12260), and SFFV-KRAB-dCas9-P2A-mCherry (Addgene ID 60954), respectively. The envelope, packaging, and transfer plasmids were transfected into Lenti-HEK cells at a ratio of 1:2:2. The supernatant was collected after 48 h and subsequently filtered using a 0.45-μm filter. The filtered viral supernatant was mixed with complete medium at a 1:1 ratio, supplemented with Polybrene (8 μg/mL), and added to HepG2 cells grown to a confluence of 60 to 70%. After an incubation of 48 h, the viral medium was replaced with growth medium (described above), and the cells were observed for mCherry signal. The mCherry-positive cells were subjected to fluorescence-activated cell sorting (FACS) (BD-Melody) in bulk, followed by another round of single-cell sorting, after which each mCherry-positive cell was allowed to grow and express stable dCas9-KRAB.

### Generation of HepG2-HBsAg and HepG2-Puro stable cell lines.

HEK-293T cells obtained from the ATCC were cultured in Dulbecco’s modified Eagle medium (Gibco) supplemented with 10% FBS (Gibco) for the generation of virus particles. The envelope and packaging plasmids used for the generation of the virus particles were pMVC-VSV-G (Addgene ID 8454) and pUMVC (Addgene ID 8449). The transfer plasmids used for making the two stable cell lines were pBABE-Puro (control cells) and HBsAg-pBABE-Puro (cloned as described above). The envelope, packaging, and transfer plasmids were cotransfected into HEK-293T cells at a ratio of 2:3:3. Cells were incubated for 48 h, after which the supernatant was collected and subsequently filtered using a 0.45-μm filter. The filtered viral supernatant was mixed with complete medium at a 1:1 ratio, supplemented with Polybrene (8 μg/mL), and added to HepG2 cells grown to a confluence of 60 to 70%. After an incubation of 48 h, the viral medium was removed, and HepG2-Puro and HepG2-HBsAg cells were selected in growth medium supplemented with puromycin (8 μg/mL) for 14 days. Constitutive expression of HBsAg in HepG2-HBsAg and HepG2-Puro cells was evaluated using the commercially available Monolisa HBsAg Ultra assay (Bio-Rad). All samples tested were within the linear range of the ELISA, as described previously (63–66). The medium from the cells was added to anti-HBs-coated microplates, which were further processed as per the manufacturer’s protocol.

### Bioinformatic analysis.

As described previously, binding sites corresponding to NF-κB factors in the LINC00665 promoter were identified using Jaspar database (http://jaspar.genereg.net/) (31, 41). We confirmed that each of these binding sites conforms to canonical κB sites having the consensus sequence 5′-GGGRNWYYCC-3′ (where N is any base, R is purine, W is adenine or thymine, and Y is pyrimidine) (42, 67) (Table S2).

The gene expression microarray data sets used in this study— GSE4549 (34), GSE84402 (35), GSE83148 (36), and GSE62232 (37)—were obtained from the GEO database (https://www.ncbi.nlm.nih.gov/geo). The series matrix files for these data sets were extracted and log_2_ transformed. Differentially expressed genes in each data set were screened using the limma (linear model for microarray analysis; Bioconductor) package in R 4.1.1 (68). Prior to the differential expression analysis, the data were filtered to retain only the 50% most variable probes. This has been shown to increase the power to detect differential expression (69). Probes with *P* values of <0.05 were regarded as differentially expressed. The *P* values were false-discovery-rate (FDR) corrected using the Benjamini-Hochberg method. The differentially expressed probes were matched to their corresponding official gene symbols to identify the differentially expressed genes and lncRNAs, using the Ensembl database (70). For multiple probes corresponding to one gene, only the probes with the most significant gene expression value were retained.

### Cell proliferation assay.

Cell proliferation was assessed using Cell Counting Kit-8 (CCK-8; Sigma, 96992) as described previously (71). Briefly, cells were grown in 96-well plates under appropriate conditions for 2, 4, or 6 days. This was followed by addition of 10 μL CCK-8 solution for 4 h, and absorbance was measured at 450 nm in a microplate reader.

### Clonogenic assay.

Transfected cells were grown for 24 h, trypsinized, and seeded in 6-well plates at 4,000 cells per well (72). Cells were grown for 2 weeks, after which medium was removed and methanol containing 0.1% (wt/vol) Coomassie blue dye was added to fix and stain adherent colonies. The plates were then imaged using a GelDoc system (Bio-Rad), and the number of colonies were determined using the ImageJ program (73).

### Scratch assay.

Cells were seeded at 80% to 90% confluence in 6-well plates and transfected with appropriate plasmids, for gain-of-function or loss-of-function studies. A sterile microtip was used to create a straight scratch in the cell monolayer, and detached cells were removed by washing with 1× phosphate-buffered saline (PBS). The wounded area was observed by phase-contrast microscopy, and percentage wound closure was calculated using ImageJ.

### Apoptosis assay.

Cellular apoptosis was measured by detecting caspase 3/7 activity (Caspase-Glo 3/7 assay; Promega), as per the manufacturer’s protocol. Briefly, cells were seeded at a density of 5,000 cells per well in a 96-well plate and grown for 48 h. We added 100 μL of the caspase reagent to each well for 1 h, after which the luminescent signal was recorded using a luminometer.

### HBV preparation and infection.

HBV infection was carried out in HepG2-hNTCP-C4 cells using HBV derived from HepG2.2.15, as described previously (59, 74, 75). Briefly, the supernatant from HepG2.2.15 cells containing infective HBV was centrifuged to remove debris and virus particles were precipitated using polyethylene glycol (PEG) 8000 and 2.3% NaCl. This precipitate was washed, pelleted, and then resuspended in medium at a ~200-fold concentration. Real-time PCR was then performed to quantitate HBV DNA and determine the concentration of HBV in the medium (see Table S3 for primers). HepG2-hNTCP-C4 cells were grown in 6-well plates and infected using HBV suspended at a concentration of 10^6^ genome equivalents per cell in infection medium (HepG2-hNTCP-C4 culturing medium additionally supplemented with 4% PEG 8000 and 2% DMSO) (76). The infection medium was removed after 24 h, after which the cells were washed and grown in culturing medium for 3 days before being harvested.

### Real-time PCR assay.

RNA was extracted using a commercially available RNA extraction kit (RNeasy minikit; Qiagen), as per the manufacturer’s protocol. Purified RNA (1 μg) was treated with DNase I, and cDNA was synthesized using the iScript cDNA synthesis kit (Bio-Rad). Real-time PCR was performed using FastStart Essential DNA Green Master (Roche), using appropriate primer pairs (Table S3). Primers for NF-κB target genes (IL1A and GPC6) were used as described previously (77).

### Luciferase assay.

Luciferase assay was performed using the commercially available dual-luciferase reporter assay system (Promega), as described previously (75, 78). Briefly, the NF-κB promoters (wild type and κB mutant) were cloned in the PGL3-basic vector as described above and cotransfected with pRL-TK (internal control reporter) into cells seeded in 24-well plates. The cells were washed and lysed as per the manufacturer’s protocol, and the luminescent signal was recorded using a luminometer (BioTek).

### ChIP.

HepG2-Puro and stable HepG2-HBsAg cells grown in T75 flasks were processed using the EZChIP kit (Millipore) according to the manufacturer’s protocol. Briefly, cells fixed with 1% formaldehyde were lysed and the obtained cell lysate was sonicated as described previously (75). The sonicated lysate was further centrifuged and supernatant containing chromatin in the range of ~200 to 1,000 bp was collected. The fragmented chromatin was precleared using protein agarose G and later incubated with anti-NF-κB1 (3035; Cell Signaling Technology), anti-RelA (8242; Cell Signaling Technology), anti-c-Rel (12659; Cell Signaling Technology) or rabbit IgG (Thermo Fisher Scientific, 02-6102) antibodies overnight at 4°C. The immunoprecipitated chromatin was purified by adding protein G-conjugated agarose beads to each sample for an hour. The protein G-agarose–antibody–chromatin complex was subjected to multiple rounds of washing and subsequently eluted in the presence of 100 mM NaHCO_3_ and 1% SDS. Finally, the DNA-protein cross-linking was reversed overnight using 5 M NaCl at 65°C, and the enriched DNA was purified using spin columns provided in the kit. Real-time PCR was performed as described above to quantitate the immunoprecipitated DNA using primers provided in Table S3.

### Immunofluorescence assay.

HepG2-Puro and HepG2-HBsAg were seeded at a concentration of 8 × 10^4^ cells per well of a 6-well plate (Corning), preimmersed with 22-mm^2^ square coverslips (Corning). The cells were grown for 48 h, after which they were washed with 1× PBS. The cells were then fixed using a fixing solution (3% paraformaldehyde, 5 μM EGTA [pH 8], 1 μM MgCl_2_) for 10 min and washed twice with washing buffer (30 μM glycine in PBS, 5 μM EGTA, and 10 μM MgCl_2_). Following this, the cells were permeabilized with a permeabilization buffer (0.2% Triton X-100 in PBS, 5 μM EGTA, and 10 μM MgCl_2_) and subsequently washed twice. Blocking was carried out for 30 min in a blocking buffer (0.5% bovine serum albumin [BSA] in PBS, 5 μM EGTA, 10 μM MgCl_2_). The samples were then incubated overnight along with the primary anti-NF-κB1 (3035; Cell Signaling Technology), anti-Rel-A (8242; Cell Signaling Technology), or anti-c-Rel (12659; Cell Signaling Technology) antibodies at a dilution of 1:500 on a rocker at 4°C. The samples were then washed three times with the washing buffer and incubated with Alexa Fluor 488 (Thermo Fisher Scientific) at a dilution of 1:500 for 2 h. Washing was carried out three times after incubation with the secondary antibody. The coverslips were then mounted on the glass slides (Corning) with a drop of Prolong Gold antifade mountant with DAPI (Thermo Fisher) and were imaged with an EVOS FL imaging system under a 60× objective. Quantification for the images was carried out using Fiji (ImageJ).

### Assessment of the stability of LINC00665 in the presence of HBsAg.

HepG2 cells were transfected with pcDNA (control) or HBsAg-pcDNA and used to assess the impact of HBsAg expression, if any, on the stability of LINC00665. After 12 h of transfection, actinomycin D was added to the cell culture medium to obtain a final concentration of 5 μg/mL. Samples were collected at 0, 8, 16, and 24 h following the addition of actinomycin D. The extracted RNA was used to assess the stability of LINC00665 using appropriate primers (Table S3) by plotting the relative abundance of the RNA at different time points using the 0-h values for normalization as described previously (79).

### Statistical analysis.

At least 3 independent experiments (*n* = 3) were performed to generate the experimental data. Statistical differences were assessed using Student’s *t* test, and *P* values of <0.05 were considered significant.

### Data availability.

All data generated or analyzed during this study are included in this article and its supplemental material.
