# Supplementary material for: Hepatitis B Virus-Encoded HBsAg Contributes to Hepatocarcinogenesis by Inducing the Oncogenic Long Noncoding RNA LINC00665 through the NF-κB Pathway
Source: Microbiol Spectr. 2022 Aug 22;10(5):e02731-21. doi: 10.1128/spectrum.02731-21 (PMC9603668; doi:10.1128/spectrum.02731-21)
Supplement: Supplemental file 1 — Supplemental material. Download spectrum.02731-21-s0001.pdf, PDF file, 0.4 MB [file spectrum.02731-21-s0001.pdf]

SUPPLEMENTARY INFORMATION

HBV-encoded HBsAg contributes to hepatocarcinogenesis by inducing oncogenic lncRNA LINC00665 through the NF-κB pathway

Shivaksh Ahluwalia[a], Belal Ahmad[a,#], Uzma Salim[a,#], Dipannita Ghosh[a,#], Vinay Kamuju[b,#], Arpita Ghosh[c,d,#], Khadija Dabeer[a,#], Manoj Balakrishna Menon[a] and Perumal Vivekanandan[a, *]

[a]Kusuma School of Biological Sciences, Indian Institute of Technology Delhi, New Delhi, India

[b]Department of Biosystems Science and Engineering, ETH Zurich, Basel, Switzerland

[c]CSIR - Institute of Genomics & Integrative Biology , Mathura Road , New Delhi, India
[d]Academy of Scientific & Innovative Research, CSIR- Human Resource Development Centre, Sector 19, Kamla Nehru Nagar, Ghaziabad, Uttar Pradesh, India

# These authors contributed equally

*Address correspondence to Perumal Vivekanandan at vperumal@bioschool.iitd.ac.in

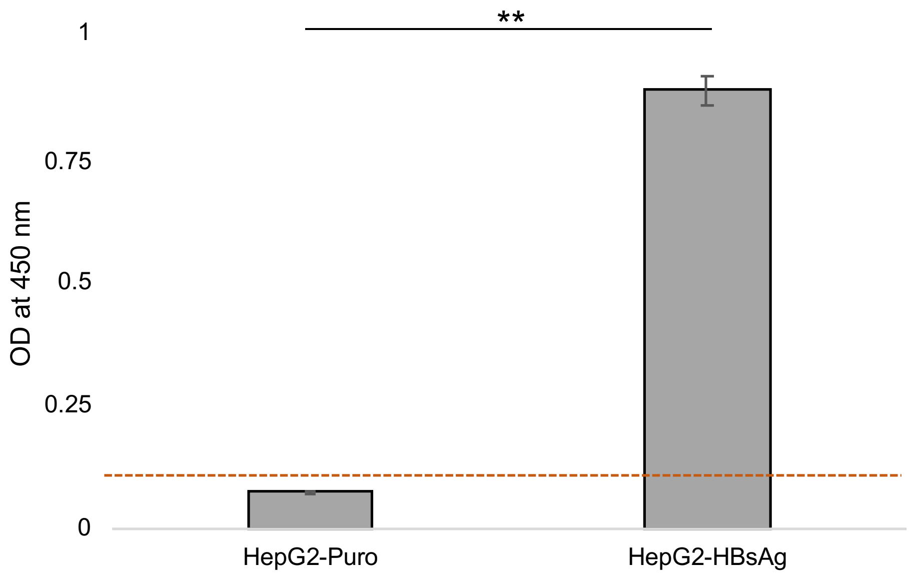

Fig. S1 HepG2-HBsAg cells constitutively express HBsAg. HepG2 cells stably expressing HBsAg were prepared using retroviral transductions as described in the methods section. The supernatant from stable HepG2-HBsAg and control HepG2-Puro cells was collected after 48 hours and secreted HBsAg was quantitated by ELISA. The cut-off was set at 0.1153 units as described in the manufacturer's protocol. A signal above the cut-off confirmed that HBsAg was produced in HepG2-HBsAg, but not in HepG2-Puro cells. All data are means ±S.D. for three independent experiments (n=3). **P<0.01, was analysed by paired Student's *t*-test.

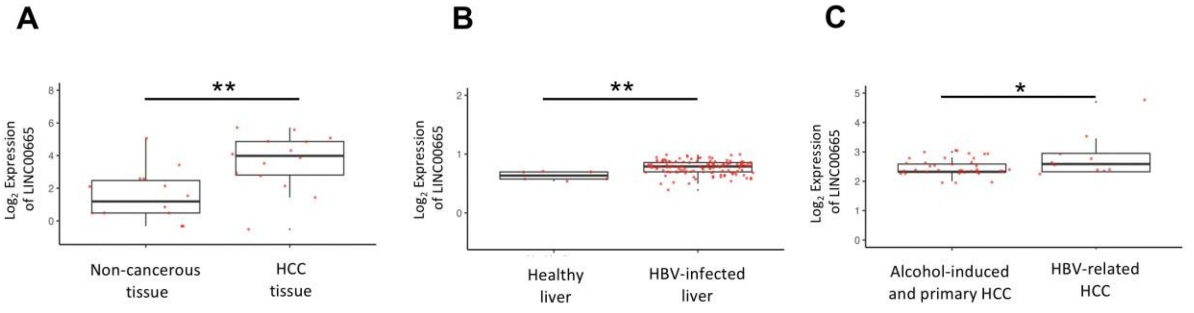

Fig. S2 LINC00665 expression is elevated in GEO datasets pertaining to liver samples from HCC (including HBV-related HCC) or HBV infected patients. Box plots depicting LINC00665 expression in gene expression datasets (A) GSE84402, (B) GSE83148 and (C) GSE62232. Please refer to Results section for details on study design. ***P*<0.01, was analysed by paired Student's *t*-test.

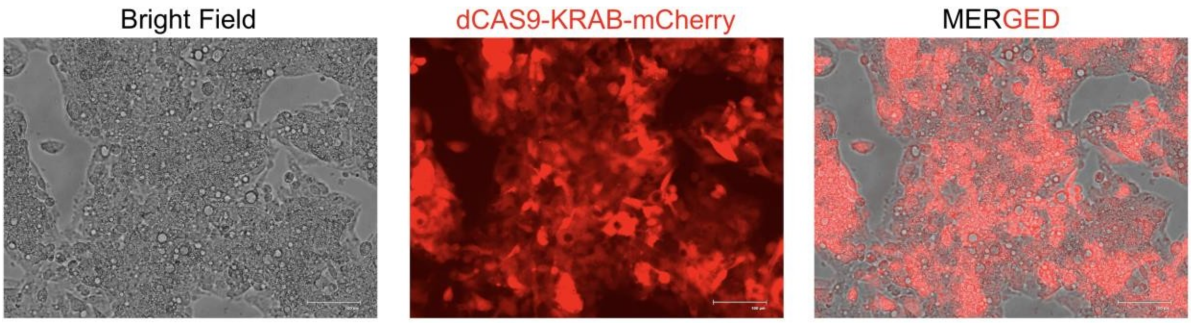

Fig. S3 HepG2 cells constitutively expressing dCas9-KRAB repressor were prepared for CRISPRi. A transfer plasmid carrying dCas9-KRAB fusion protein and mCherry fluorescent protein was used for preparation of lentivirus particles for HepG2 transduction, as per the details described in the methods section. Transduced HepG2 cells were FACS sorted and stable expression of these proteins was confirmed by fluorescent microscopy. Scale bar 100 μm.

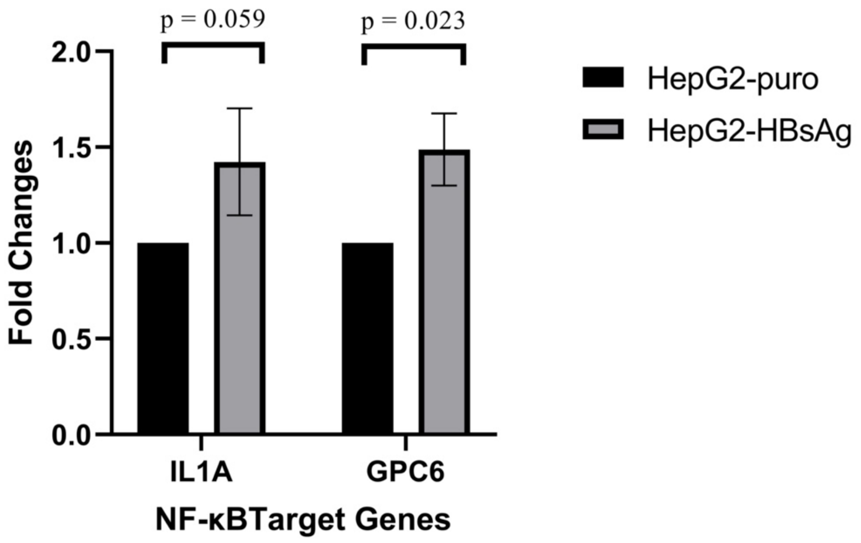

Fig. S4 Effect of HBsAg expression on NF-κB target genes. HepG2-HBsAg and control HepG2-Puro cells grown for 48 hours and NF-κB target genes (IL1A and GPC6) were assessed using qPCR. The fold change in NF-κB target genes is normalized to their expression in the control HepG2-Puro cells and GAPDH was used as the house-keeping gene. Student's t-test was used for statistical analysis.

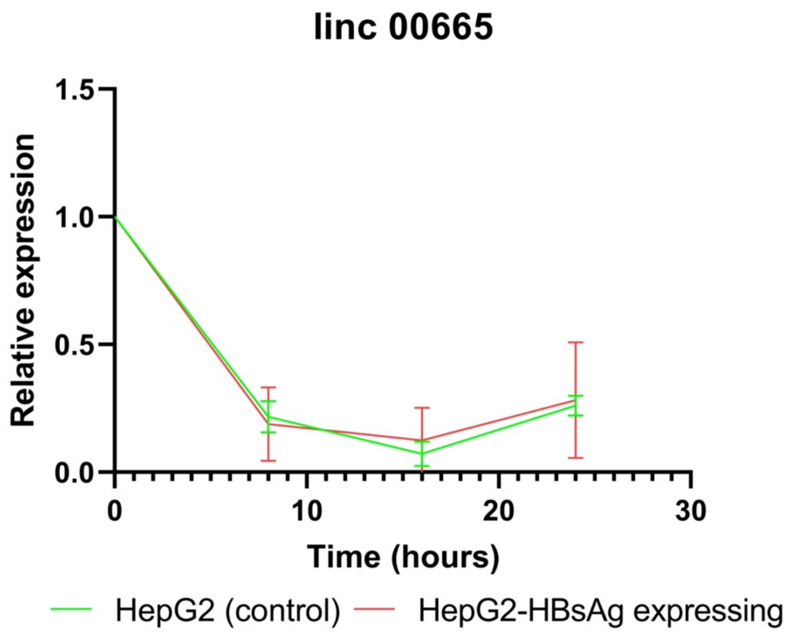

Fig. S5 HBsAg expression does not alter the stability of LINC 00665. HepG2 cells transiently transfected with pcDNA (empty vector) or HBsAg-pcDNA (HBsAg-expression construct). After 12 hours of transfection, Actinomycin D stock was added to obtain a final concentration of 5 µg/ml in culture media. Samples were collected at 0, 8, 16, and 24 h time points following Actinomycin D addition. Real-time RT-PCR was used to measure the stability of LINC00665. The expression of HBsAg did not alter the stability of linc00665.

Table S1 gRNA target sequences in the LINC006665 promoter. The indicated sequences were targeted in the LINC00665 promoter using sgRNA expressed from MLM3636 expression vector in HepG2-dCas9-KRAB

**gRNA**	**Target Sequence**
LINC00665 sgRNA 1	GGCCGAATAGCCCAAGACTGAGG
LINC00665 sgRNA 2	GAAATTGGCCATTGCCACGGCGG
LINC00665 sgRNA 3	GCGCAAGGTTGGTCTGCAGGTGG
LINC00665 sgRNA 4	TGCCGGACATGGGACGCTGGAGG

Table S2 – Sequence and position of κB sites relative to the TSS in the LINC00665 minimal promoter, along with mutations used to disrupt the κB sites.

S. No.	Position (TSS)	Strand	Predicted Sequence	Mutated Sequence
1	-278	-	GGAGATTTCA	TCAGGTATCA
2	-277	-	CGGAGATTTC	CTCAGGTATC
3	-164	-	GCGGGCTTCC	TCAGGAAAGC
4	111	+	TGTAGTTTCC	TATGGCCTGA
5	111	+	TGTAGTTTCC	TATGGCCTGA
6	111	+	TGTAGTTTCCT	TATGGCCTGAT
7	162	+	GGGGCTTTTTC	GTTTCTCACCA
8	163	+	GGGCTTTTTC	TTTCTCACCA
9	163	+	GGGCTTTTTCA	TTTCTCACCAA

Table S3 List of primers and their sequences used in this study. For details please refer to methods section. FP - Forward Primer, RP - Reverse Primer

Name	FP or RP	Sequence
HBsAg cloning in pBABE	FP	TATAGAATTCATGGAGAACATCACATCAGG
	RP	TATAGTCGACTTAAATGTATACCCAAAGACAAAAG
HBsAg cloning in pcDNA	FP	TATAGGTACCATGGAGAACATCACATCAGG
	RP	TATACTCGAGTTAAATGTATACCCAAAGACA
LINC00665 qPCR	FP	CAGCTTGTAGGGGGGAGTGC
	RP	ATCGTGATGACGGTGTGAGG
NR2F2-AS1 qPCR	FP	GCTTTGCTAGCTCCTCATGC
	RP	GACAACTGGCTCCCTCATCT
LINC00242 qPCR	FP	AGAACCTTCCCGGCATTGAG
	RP	GAAGTGAGGAACAGGGACCG
UBAC2-AS qPCR	FP	CAGGAGCGCCACTTCTAATC
	RP	TGAGTGACCAGATCCCACTG
Lnc-APOA4-1 qPCR	FP	CCGTGTCCTCCTGCTGTTATC
	RP	CTGGTTCAGAGTCTGTCATGAGG
BANCR qPCR	FP	TGCTGAGAAGTTCAGAGTCAAAC
	RP	GCCAGGGATGACTTGCGTAT
LINC00470 qPCR	FP	GCATGAAAAGTGACTGGATTTGT
	RP	GACTGGCCGATGTCAATTTCA
TEX41 qPCR	FP	CCCTTCGAGTAACACCCACA
	RP	CCCCTGGGTCTCACAGGATA
GAPDH qPCR	FP	TGCACCACCAACTGCTTAGC
	RP	GGCATGGACTGTGGTCATGAG
HBV DNA qPCR	FP	CTTCATCCTGCTGCTATGCCT
	RP	AAAGCCCAGGATGATGGGAT
LINC00665-sgRNA 1 cloning in MLM3636	Sense	ACACCGGCCGAATAGCCCAAGACTGG
	Anti-Sense	AAAACCAGTCTTGGGCTATTCGGCCG
LINC00665-sgRNA 2 cloning in MLM3636	Sense	ACACCGAAATTGGCCATTGCCACGGG
	Anti-Sense	AAAACCCGTGGCAATGGCCAATTTCG
LINC00665-sgRNA 3 cloning in MLM3636	Sense	ACACCGCGCAAGGTTGGTCTGCAGGG
	Anti-Sense	AAAACCCTGCAGACCAACCTTGCGCG
LINC00665-sgRNA 4 cloning in MLM3636	Sense	ACACCGTGCCGGACATGGGACGCTGGG
	Anti-Sense	AAAACCCAGCGTCCCATGTCCGGCACG
IL1A qPCR	FP	ACTGAGGTCCCAGAAAC
	RP	TTGAAACAAGAAGCCAC
GPC6 qPCR	FP	CTAAACAGAGGCTAAAGT
	RP	TAAAGCAGGTGATACAAG
